# Prasugrel switching from clopidogrel after percutaneous coronary intervention for acute coronary syndrome in Taiwanese patients: an analysis of safety and efficacy

**DOI:** 10.1007/s12928-021-00771-w

**Published:** 2021-04-04

**Authors:** Ping-Yen Liu, Cheng-Huang Su, Feng-Yu Kuo, Wen-Lieng Lee, Yi-Chih Wang, Wei-Shiang Lin, Pao-Hsien Chu, Tse-Min Lu, Ping-Han Lo, Cheng-Han Lee, Wei-Ren Lan, Chien-Lung Huang, Shuji Tsukiyama, Wei-Chen Yang, Li-Chung Cheng, Virginia Rafael, Christian Nikolajsen, Wei-Hsian Yin

**Affiliations:** 1 Department of Cardiology, National Cheng Kung University Hospital, College of Medicine, National Cheng Kung University, Tainan, Taiwan; 2 Institute of Clinical Medicine, College of Medicine, National Cheng Kung University, Tainan, Taiwan; 3 Cardiovascular Center, Department of Internal Medicine and Medical Research, Mackay Memorial Hospital, Taipei, Taiwan; 4 Mackay Medical College, New Taipei City, Taiwan; 5 Division of Cardiology, Department of Medicine, Kaohsiung Veterans General Hospital, Kaohsiung, Taiwan; 6 Division of Interventional Cardiology, Cardiovascular Center, Taichung Veterans General Hospital, Taichung, Taiwan; 7 Division of Cardiology, Department of Internal Medicine, National Taiwan University College of Medicine and Hospital, Taipei, Taiwan; 8 Division of Cardiology, Tri-Service General Hospital, National Defense Medical Center, Taipei, Taiwan; 9 Division of Cardiology, Department of Internal Medicine, Chang Gung Memorial Hospital at Linkou, Chang Gung University College of Medicine, Taoyuan, Taiwan; 10 Division of Cardiology, Department of Medicine, Taipei Veterans General Hospital, Taipei, Taiwan; 11 Healthcare & Service Center, Taipei Veterans General Hospital, Taipei, Taiwan; 12 Faculty of Medicine, School of Medicine, National Yang Ming University, Taipei, Taiwan; 13 Division of Cardiology, Department of Internal Medicine, China Medical University Hospital, China Medical University, Taichung, Taiwan; 14 Division of Cardiology, Heart Center, Cheng Hsin General Hospital, Taipei, Taiwan; 15 Daiichi Sankyo Co. Ltd., Chuo-ku, Tokyo, Japan; 16 Daiichi Sankyo Taiwan Ltd., Taipei, Taiwan; 17 Linical, Madrid, Spain

**Keywords:** Acute coronary syndrome, Antiplatelet, Prasugrel, Regimen switch, P2Y12 reaction unit

## Abstract

The recommended maintenance dose of prasugrel for East Asian populations (i.e., Japanese and Taiwanese) is 3.75 mg as part of dual antiplatelet therapy (DAPT) for the prevention of recurrent ischemia and stent thrombosis in acute coronary syndrome (ACS). This modified dosage regimen has been established in studies conducted in Japan; however, the efficacy and safety of switching from clopidogrel to prasugrel DAPT among Taiwanese patients remain to be explored. In this phase IV, multicenter, single-arm, open-label study, we evaluated the 4-week pharmacodynamic response, and the 48-week safety outcomes of prasugrel 3.75 mg after a switch from clopidogrel in Taiwanese ACS patients. A total of 203 prasugrel-naïve ACS patients (over 90% male) who had received post-PCI clopidogrel DAPT for at least 2 weeks were enrolled from ten medical centers in Taiwan and subsequently switched to prasugrel 3.75 mg DAPT. Four weeks after the switch, P2Y12 reaction unit (PRU) values were significantly decreased in the total cohort (mean − 18.2 ± 48.1; 95% confidence interval − 24.9 to − 11.5, *p* < 0.001), and there was an overall consistent antiplatelet response in the treated subjects. The proportion of patients with high on-treatment platelet reactivity (HPR; PRU > 208) dropped from 23.5 to 10% (*p* < 0.001). Female sex was associated with a greater PRU reduction with prasugrel, whereas HPR at baseline, age ≥ 65 years, and body mass index ≥ 25 best predicted HPR at Week 4. Throughout the 48-week treatment with prasugrel, the incidences of MACE (1.0%) and TIMI major bleeding (2.0%) were rather low, accompanying an acceptable safety profile of TIMI minor (6.4%) and non-major, non-minor clinically relevant bleeding (3.0%). Overall, switching to the maintenance dose of prasugrel (3.75 mg) was observed to be effective and well tolerated among post-PCI ACS patients in Taiwan. Clinical Trial Registration Number: NCT03672097.

**Supplementary Information:**

The online version contains supplementary material available at 10.1007/s12928-021-00771-w.

## Introduction

Although both clopidogrel and prasugrel effectively reduce the ischemic risk in patients with acute coronary syndrome (ACS) undergoing percutaneous coronary intervention (PCI), clopidogrel is well known for its highly variable platelet inhibitory effect and delayed onset. Prasugrel, a newer generation thienopyridine P2Y12 inhibitor, demonstrates a more predictable antiplatelet activity and has a rapid onset of action, which may translate to its clinical stability and safety [1, 2]. Switching between clopidogrel and prasugrel (or other potent P2Y12 inhibitors) may be considered in certain situations (i.e., diminished clinical effects or change in risk strata, *CYP2C19* loss-of-function mutations, compliance issues, adverse events, cost, and drug availability) [3]. The pharmacodynamic impact of switching from clopidogrel to prasugrel was first documented in the SWitching Anti Platelet (SWAP) study, in which 1-week maintenance dose of prasugrel (10 mg) switched from maintenance dose of clopidogrel was associated with a further reduction in platelet reactivity [4]. Such impact was later revisited by a Japanese randomized trial of 136 patients with ACS who underwent PCI with elective stenting—switching from maintenance dose of prasugrel (3.75 mg) to clopidogrel significantly increased mean P2Y12 reaction unit (PRU) values compared with continuing on prasugrel [5]. Likewise, a study in Japanese patients with stable coronary artery disease (CAD) showed a significant increase in platelet inhibition after switching from clopidogrel to the maintenance dose of prasugrel (3.75 mg) [6]. Although there is consistency in reduction of platelet reactivity among Japanese ACS and stable CAD patients, more studies are needed to establish whether this benefit translates to protection against cardiovascular events in other East Asian populations.

Prasugrel is currently recommended by Japanese Guideline (Class I) [7, 8] at a dose of 3.75 mg daily, which was established after global and Japanese pivotal studies. In TRITON-TIMI-38, standard-dose prasugrel exhibited greater platelet inhibition in East Asians than Caucasians [2]. The PRASFIT-ACS study in Japan tested this personalized prasugrel dose, showing that the efficacy (23% risk reduction in MACE at 24 weeks) and safety of maintenance dose prasugrel (3.75 mg) were similar to those of maintenance dose clopidogrel (75 mg) [9]. However, the pharmacodynamic response, net clinical benefit, and overall appropriateness of 3.75 mg daily prasugrel await formal investigation in Taiwanese patients.

In this phase IV study, we aimed to clarify the short-term (Period 1; 4 weeks) and long-term (Period 2; 28 weeks and an optional extension to 48 weeks) efficacy and safety of 3.75 mg daily prasugrel after a switch from clopidogrel (75 mg daily) in 204 prasugrel-naïve patients with ACS during the post-PCI dual antiplatelet therapy (DAPT) maintenance phase.

## Materials and methods

### Study design

The Switch Study was a phase IV, multicenter, single-arm, open-label, prospective study conducted in Taiwan to determine the efficacy and safety of switching from clopidogrel (75 mg daily) to prasugrel (3.75 mg daily) during the maintenance phase. Study enrollment took place at ten Taiwanese medical centers between September 2018 and November 2019.

The Switch Study protocol was approved by the institutional ethics committees of each study site before study initiation. This study was conducted in compliance with the International Council for Harmonization of Technical Requirements for Pharmaceuticals for Human Use Harmonized Tripartite Guideline for Good Clinical Practice, the local laws and regulations of Taiwan, and the Declaration of Helsinki.

### Study population

We first screened patients of both sexes of any ethnicity with recently diagnosed ACS (either non-ST elevation myocardial infarction [NSTEMI], ST elevation myocardial infarction [STEMI], or unstable angina [UA]) who had been treated with PCI and one of the following DAPT regimens [10, 11]:

- Clopidogrel loading dose (LD; 300 or 600 mg) at the time of PCI, followed by clopidogrel maintenance dose (75 mg daily) and aspirin (81–100 mg daily) for 2–8 weeks.
- Ticagrelor LD (180 mg) at the time of PCI, followed by maintenance dose of clopidogrel (75 mg daily) and aspirin (81–100 mg daily) for 2–8 weeks.
- Ticagrelor LD (180 mg) at the time of PCI, followed by maintenance dose of ticagrelor (90 mg twice daily) and aspirin (81–100 mg daily) for 1–4 weeks, then switched either directly or via clopidogrel LD (300 or 600 mg) to maintenance dose of clopidogrel (75 mg daily) and aspirin (81–100 mg daily) for 2–4 weeks.
- Based on investigators’ judgment, a maximum 8-week period of P2Y12 inhibitor maintenance dose where prasugrel was not allowed, followed by the continual use of maintenance dose of clopidogrel and aspirin (81–100 mg daily) for ≥ 2 weeks.

We included patients ≥ 20 years of age, weighing ≥ 50 kg at the time of screening, and who provided signed informed consent. We excluded patients who had active bleeding, significantly increased risk of hemorrhage, or a history of stroke within 3 months of the informed consent date; allergies or hypersensitivity to the study drugs; significant comorbidities (i.e., end-stage renal disease) including but not restricted to severe hepatic disease, severe left ventricular systolic dysfunction, or significant hypertension; pregnant women and women of childbearing potential; and any other clinical or laboratory results that were judged detrimental or compromising by the investigator.

### Study procedures

Patients signed an informed consent, were screened and surveyed for previous medical histories, concomitant diseases and medications. On Day 1, eligible patients discontinued clopidogrel, switching to the trial regimen comprising daily doses of prasugrel (3.75 mg) and aspirin (81–100 mg) for 28 consecutive weeks (Day 1–196). During the treatment course, patients were obliged to attend four study visits: treatment initiation (Day 1 in Week 1); end of study Period 1 (Day 28 in Week 4); end of Week 16 (Day 112 in Week 16); and end of study Period 2 (Day 196 in Week 28; Fig. S1). In addition, an optional final visit was scheduled at Week 48 for a routine 1-year safety follow-up of patients’ post-PCI care. Immediately before taking the study drugs at Day 1 and Day 28, all enrolled patients underwent blood sampling, from which their PRU and high on-treatment reactivity (HPR) status were determined. All adverse events (AEs) were documented during the entire study period.

The primary endpoint was the mean change in PRU from baseline (Day 1) to the end of the 4-week prasugrel maintenance dose treatment (Week 4). The secondary efficacy endpoint was the percentage of patients with HPR (defined as PRU > 235 per protocol) by the end of Week 4. The primary safety endpoint was the incidence of non-CABG-related TIMI major bleeding in the 28-week (or optionally 48-week) prasugrel MD treatment period (Period 2); the secondary safety endpoints were the incidences of all-cause deaths, major adverse cardiac events (MACE), bleeding (major, minor, and clinically relevant bleeding events defined by the TIMI criteria), and any other AE occurring by the end of Week 4. All patients who completed Period 1 analysis remained eligible for the 24-week (or optionally 48-week) follow-up, at the end of which patients were primarily evaluated for bleeding events by TIMI criteria, as well as MACE, death and other AEs. Bleeding events were also classified by Bleeding Academic Research Consortium [BARC] criteria to extrapolate the study results to data from other investigators. The ARC-HBR criteria, an identifier of high bleeding risks based on characteristics of ACS patients at the time of PCI, was adopted for exploratory analyses in patient characteristics and safety outcomes [12–14].

### PRU testing

PRU was assessed with the VerifyNow^®^ system (Accriva Diagnostics, San Diego, CA), the method of which has been published previously [15]. Based on patients’ PRU values at baseline and the end of each study period, the percentage of patients who had HPR was calculated (defined as PRU > 235 per protocol; PRU > 208 was used in the exploratory analysis) [16–18].

### Statistical analysis

Based on the PRU results from a prior study [19], we postulated that the PRU reduction of switching from clopidogrel to maintenance dose of prasugrel (3.75 mg) is 30 (with a standard deviation [SD] of 70), and that a sample size of 200 would be needed to generate 170 completed patients under an expected drop-out rate of 15%, enabling the detection of mean PRU difference between baseline and Week 4, with a two-sided significance level of 0.05 (*α*) and an 80% power.

Descriptive analyses were applied for baseline characteristics using means and SD for continuous variables, and frequencies and percentages for categorical variables.

The primary analyses were the PRU values at and the PRU differences between baseline and Week 4. These were described by the number of analyzed cases, means, SD, and the 95% confidence intervals (CIs) in both total and prespecified subgroups. The secondary analysis was HPR at baseline and Week 4, which was expressed as frequencies/percentages. Both primary and secondary analyses were tested for statistical significance. For primary endpoint, *t* tests and Wilcoxon signed-rank tests were used where appropriate. For secondary endpoints, McNemar’s test was applied for comparing PRU values.

We adopted an analysis of covariance (ANCOVA) model and a multivariate logistic regression model to explore factors associated with change from baseline PRU values and with HPR at Week 4 (PRU > 208 as the exploratory cutoff), respectively. In both models, factors were step-wisely selected from the following variables: age, sex, body mass index, compliance, diabetic status, lipid disorder status, and decreased GFR status (eGFR < 60). A probability of < 0.2 during the stepwise ANCOVA further determined the entering and staying of a variable in the model. For factors selected into the final models, adjusted least squares (LS) means with 95% CI, or adjusted odds ratios (ORs) with 95% CI were shown along with the respective *p* values. Statistical analyses were performed using SAS^®^ version 9.4.

The primary safety endpoint, non-CABG-related TIMI major bleeding for 28 weeks (or optionally up to 12-month P2Y12 inhibitor treatment period after PCI) was evaluated using Kaplan–Meier estimates. Secondary safety endpoints were the incidences of events (major, minor, clinically relevant bleeding, and major adverse cardiovascular events) during the 4-week maintenance dose of prasugrel (3.75 mg) treatment (Period 1), and the incidences of events (minor and clinically relevant bleeding events and MACEs) during the 28-week (or optionally 48-week) maintenance dose of prasugrel (3.75 mg) (Period 2). Additional safety endpoints included the incidences of AEs and deaths were also recorded. Bleeding events by Bleeding Academic Research Consortium (BARC) classification were also included in the exploratory analysis. Major and minor bleeding events according to BARC criteria were analyzed and summarized as pre-treatment AEs and treatment-emergent AEs (TEAEs) for Study Period 1 and Period 2. The incidence of BARC type 2, 3 or 5 bleeding was analyzed using Kaplan–Meier estimates, and the association between these events and prognostic factors was analyzed using a multivariate logistic model. The association between the presence of at least one net adverse clinical event (MACEs + BARC type 2, 3 or 5 bleeding) and prognostic factors was evaluated using a multivariate logistic model. Additional safety analyses by prespecified subgroups included the incidences of TIMI major bleeding, BARC type 2, 3 or 5 bleeding and MACEs using Kaplan–Meier estimates, by HPR (PRU ≥ 208 versus PRU < 208) at Week 4, and their Academic Research Consortium-High Bleeding Risk (ARC-HBR) (HBR versus non-HBR) status.

## Results

### Patient demographics

**Fig. 1:**
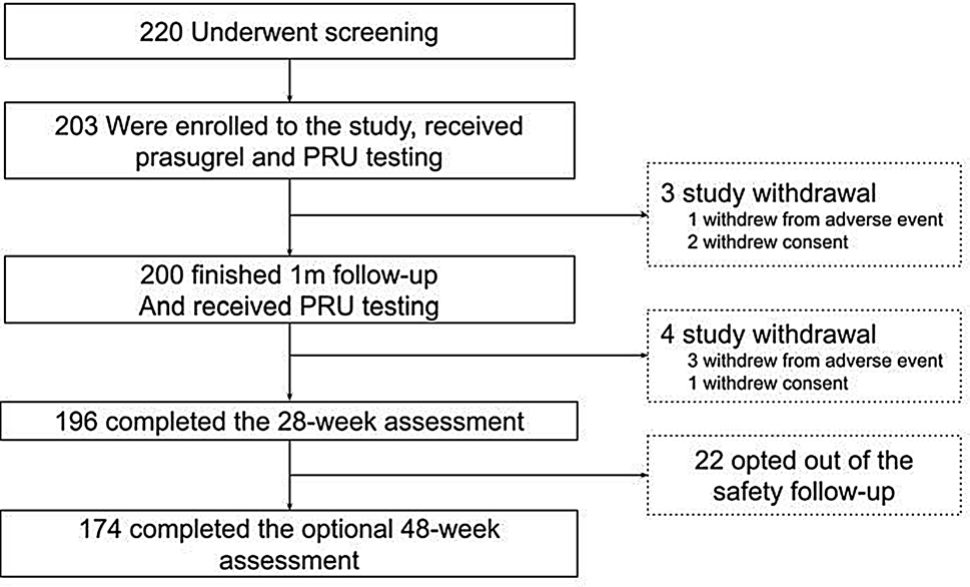
Patient enrollment flowchart for Switch Study (NCT03672097) showing the flow of patients enrolled from the start of the study to end of Period 1 and Period 2. The number of patients and the reasons for patients not available for analysis are indicated. *1m* 1 month, *PFT* platelet function testing

Among the 203 patients who were enrolled and received treatment, 200 completed the 4-week initial trial (Period 1). Three withdrew informed consent and one withdrew because of a treatment-emergent AE (TEAE; Fig. 1); 196 (96.1%) finished the 24-week assessment; 174 (85.3%) completed the optional 48-week evaluation. The completion rates were high across visits with a median of 44 weeks of treatment with prasugrel and an overall 97.9% treatment compliance. Most enrolled patients completed the 28-week (96.1%) and 48-week (85.3%) earmarks of study Period 2. In study Period 2, the mean duration of prasugrel treatment lasted for mean 41.1 ± 8.9 weeks.

The enrolled patient cohort had a mean age of 60.6 ± 10.0 years, were predominantly male (90.6%), and all Asian in ethnicity (Table 1). Patients with STEMI, NSTEMI, and UA represented 35.5, 33.5, and 31.0% of the total cohort, respectively. Most patients (89.7%) received drug-eluting stents, whereas the remaining received bare metal stents. The top three comorbidities were lipid disorders, hypertension, and diabetes, which were reported in 73.4, 59.1, and 34.5% of patients, respectively. Patients’ prevalent uses in statin (85.2%) and beta-blocker (62.1%) were consistent with current clinical practice, whereas the proportions of patients receiving proton pump inhibitors (22.2%) and calcium channel blockers (18.7%) were relatively low. There were minimal pre-treatment bleeding and ischemic event rates — only 1.5% had clinically relevant bleeding while there were no reports of MACE (Table S1).

**Table 1:** Baseline characteristics of total and *CYP2C19* genotyped patients in the Switch Study

Characteristics	All (*N* = 203)
Age, years	60.6 (10.0)
≥ 65 years	73 (35.9)
Sex (male)	184 (90.6)
Asians	203 (100)
BMI, kg/m^2^	26.2 (3.5)
Systolic blood pressure, mmHg	126.9 (16.0)
Heart rate, bpm	75.3 (10.8)
UA	63 (31.0)
NSTEMI	68 (33.5)
STEMI	72 (35.5)
PCI stent types—DES	182 (89.7)
Medical history	
Diabetes	70 (34.5)
Lipid disorders	149 (73.4)
Hypertension	103 (50.7)
Prior stroke	4 (2.0)
Previous AMI	5 (2.5)
Concomitant drugs	
Statins	173 (85.2)
Beta-blockers	126 (62.1)
Proton pump inhibitors	53 (26.1)
Calcium channel blockers	30 (14.8)
Hemoglobin, g/dL	14.1 (1.4)
< 11	4 (2.0)
11–13 (or 11–12 in female)	35 (17.2)
> 13 (or > 12 in female)	164 (80.8)
Platelet count, 10^9^/L	229.5 (65.0)
ARC-HBR	22 (10.8%)

Continuous variables are shown in mean (standard deviation); categorical variables are shown in number of patients (% of total patients)

*AMI* acute myocardial infarction, *BMI* body mass index, *bpm* beat per minute, *DES* drug-eluting stent, *N* number of patients, *NSTEMI* non-ST elevation myocardial infarction, *PCI* percutaneous coronary intervention, *STEMI* ST elevation myocardial infarction, *UA* unstable angina

In terms of P2Y12 inhibitor prescription patterns before switching to prasugrel, 54.2% received a loading dose of ticagrelor while 35.5% received clopidogrel. For maintenance, 63.1% received clopidogrel while 36.9% received ticagrelor. There were 10.3% of patients who did not receive a loading dose of P2Y12 inhibitors at PCI. In addition, as many as 88.7% of patients stayed on prasugrel treatment for more than 28 weeks, and as few as 1.5% of patients withdrew prasugrel treatment within the first 4 weeks.

Lastly, using the ARC-HBR criteria, we identified 10.8% of patients as high bleeding risks (HBR) and 85.2% as non-HBR.

### Efficacy endpoints

**Fig. 2:**
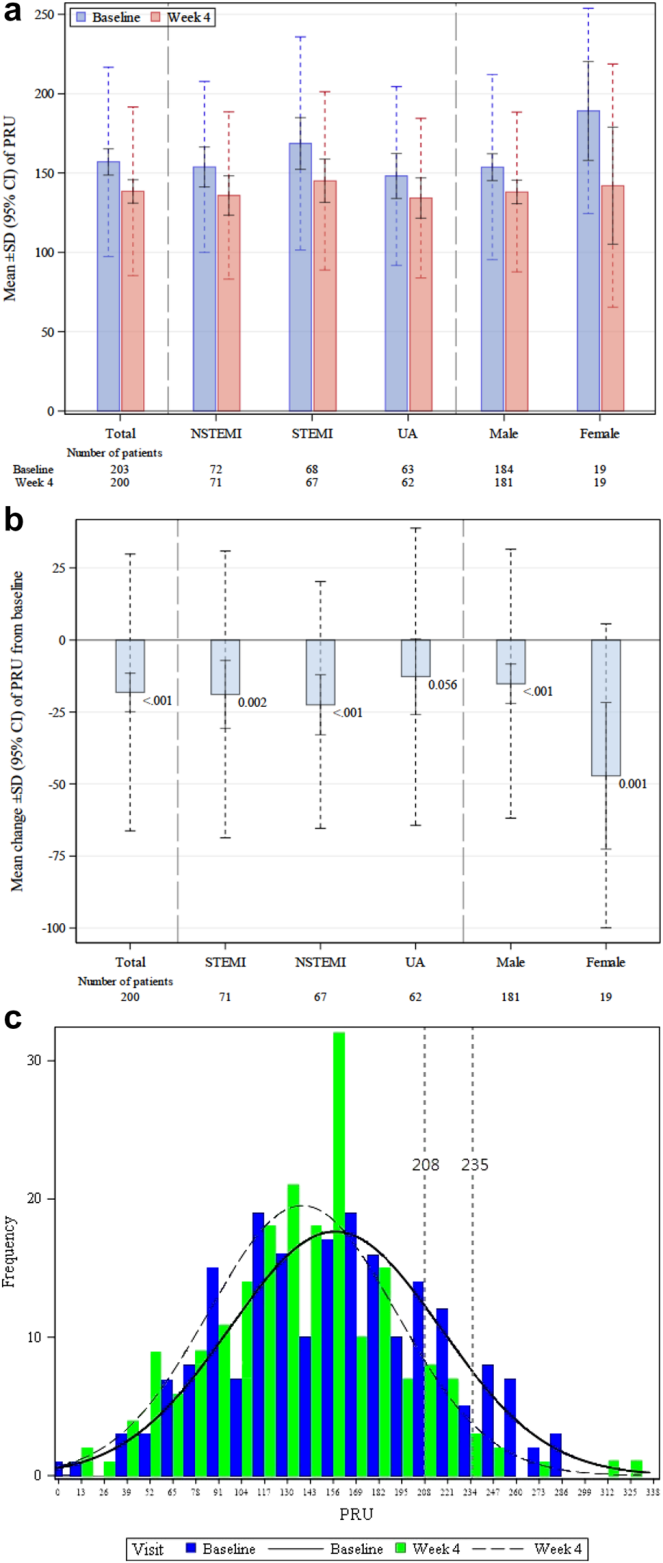
Comparisons of PRU values at baseline and Week 4 in all patients and prespecified patient subgroups. **a** PRU values at baseline and Week 4 by total patients and subgroups of ACS type and sex. **b** Change in PRU values from baseline by total patients and subgroups of ACS type and sex; *p* values of each PRU change are shown next to the corresponding data point. Each bar shows the mean PRU or change with SD (solid line) and 95% CI (dashed line). **c** Histograms of P2Y12 reaction unit (PRU) at baseline and Week 4. The dashed lines indicate the PRU levels evaluated in the study. *ACS* acute coronary syndrome, *CI* confidence interval, *NSTEMI* non-STEMI, *PRU* P2YP12 reaction unit, *SD* standard deviation, *STEMI* ST elevation myocardial infarction, *UA* unstable angina

After 4 weeks of prasugrel maintenance treatment, the mean change from baseline PRU was − 18.2 ± 48.1 (95% CI − 24.9 to − 11.5; *p* < 0.001; Fig. 2a); the PRU reductions were statistically significant in both the patients with STEMI (− 18.9 ± 49.7; *p* = 0.002) and those with NSTEMI (− 22.5 ± 42.8; *p* < 0.001) rather than in those with UA (− 12.7 ± 51.6; *p* = 0.056). The reported mean baseline PRU levels were 153.8 ± 53.8 in STEMI, 168.6 ± 67.6 in NSTEMI and 149.3 ± 56.6 in UA patients. PRU decreases were significant in both male and female patients (− 15.2 ± 46.7; *p* < 0.001 and − 47.2 ± 52.8; *p* = 0.001, respectively; Fig. 2b).

At baseline, the distribution of patient PRUs assumed a wide-based bell-shaped curve with a median of 160 and an interquartile range (IQR) of 88. After 4 weeks of treatment, the graph was shown to be narrower, with a median PRU of 142 and an IQR of 67, indicating the pharmacodynamic effects of the shift from clopidogrel to prasugrel among treated patients (Fig. 2c).

Accordingly, the percentage of patients with HPR, as defined by PRU > 235 per protocol, significantly decreased from 11.3% at baseline to 3.0% by the end of Period 1 (*p* < 0.001; Table S1). To facilitate the exploratory risk factor analysis, a more stringent PRU cutoff (PRU > 208) was utilized. The result remained consistent at a cutoff PRU of 208—where the percentage of patients with HPR dropped from 23.5% at baseline to 10.0% by the end of Period 1 (*p* < 0.001).

**Fig. 3:**
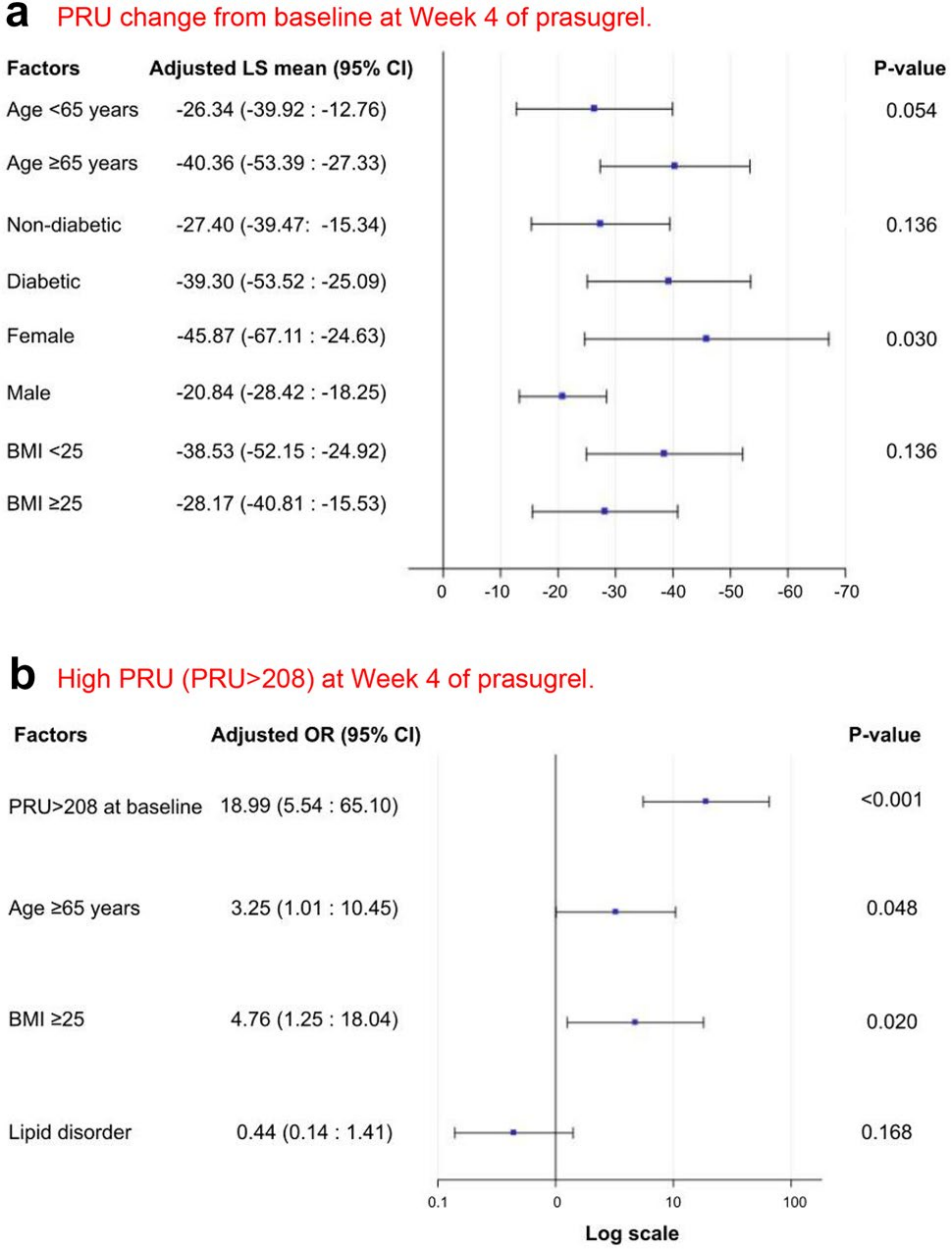
Risk factor analyses for PRU change from baseline and HPR (PRU > 208) at Week 4. **a** The adjusted LS means with 95% CI and the corresponding p values of factors associated with PRU change from baseline are shown; statistical analysis performed by a stepwise ANCOVA model. **b** The adjusted OR with 95% CI and the corresponding *p* values of factors associated with HPR (PRU > 208) at Week 4 are shown; statistical analysis performed by a multivariate logistic model. *ANCOVA* analysis of covariance, *BMI* body mass index, *CI* confidence interval, *LS* least squares, *OR* odds ratio, *PRU* P2YP12 reaction unit, *SD* standard deviation

A multivariate ANCOVA revealed that female sex was significantly associated with greater PRU reduction (LS mean = − 45.87; *p* = 0.030; Fig. 3a). Three factors with at least a 0.2 probability were selected in the ANCOVA model yet failed to reach statistical significance, namely age ≥ 65 (*p* = 0.054), diabetic status (*p* = 0.091), and BMI < 25 (*p* = 0.136). With the multivariate logistic regression model, we identified that HPR (PRU > 208) at baseline best predicted HPR (PRU > 208) at Week 4 (adjusted OR = 18.99; *p* < 0.001). In addition, BMI ≥ 25 (adjusted OR = 4.76; *p* = 0.020) and age ≥ 65 (adjusted OR = 3.25; *p* = 0.048) were also significant predictors of HPR at Week 4 (Fig. 3b).

### Safety endpoints

Notably, four patients (2%) had at least one TIMI major bleeding event during Period 2 with an increase in cumulative incidences from 0.5% at Week 28 to 2.5% at Week 48. Thirteen patients (6.4%) reported at least one treatment-emergent TIMI minor bleeding event; six (3.0%) had at least one treatment-emergent, non-major, non-minor, clinically relevant bleeding event, and two (1.0%) reporting treatment-emergent MACEs (non-fatal myocardial infarctions). The cumulative incidences at Week 48 were 7% for TIMI minor bleeding; 3.1% for non-major, non-minor clinically relevant bleeding; and 1.1% for MACE.

Among all patients, 2.5% of patients reported BARC type 3 and 5 bleeding; 5.9% reported any BARC type 2 bleeding; and 7.9% reported any BARC type 2, 3, and 5 bleeding. There were numerically more BARC type 2, 3, and 5 bleeding events in non-HPR patients at Week 48 (8.9%) versus HPR patients (0%), but the difference in cumulative incidences between the HPR subgroups was not statistically significant (*p* = 0.1846, log-rank test). There were similar rates of BARC type 2, 3, and 5 bleeding in the high bleeding risk (HBR) subgroup (9.1%) versus non-HBR (7.7%) patients and no difference in cumulative incidences between HBR subgroups (*p* = 0.8239, log-rank test). All four cases with TIMI major bleeding events were found in non-HBR patients.

## Discussion

This is the first study to demonstrate the antiplatelet efficacy and safety of switching from clopidogrel to maintenance dose of prasugrel (3.75 mg) among Taiwanese ACS patients who have undergone PCI. The Switch Study population is comparable to prior Japanese ACS cohorts reported in literature [9]. In addition, our study population is similar to the largest Taiwanese ACS cohort—the STENT-Registry (where patients received more than 9 months of DAPT)—in age, sex, and BMI, but the two cohorts varied in the proportions of UA and STEMI patients (Switch study vs. STENT registry: mean age, 60.7 vs. 60.8 years; males, 90.2% vs. 84.0%; mean BMI, 26.1 vs. 25.9; proportion of UA, 31.4 vs. 14.0%; proportion of STEMI: 35.3 vs. 54.2%) [20]. Compared with the PRASFIT-ACS cohort, the Switch Study population is slightly younger, more male predominant, with higher BMI, and with fewer STEMI (Switch Study vs. PRASFIT-ACS: mean age, 60.6 vs. 68.0 years; males, 90.2% vs. 78.2%; mean BMI, 26.1 vs. 24.2; STEMI, 35.3% vs. 49.6%); the comorbidity profile of the Switch Study population is similar to that of PRASFIT-ACS, albeit with lower prevalence of major comorbidities such as diabetes, lipid disorders, and hypertension [9]. The younger age and the predominant use of drug-eluting stents in the Switch Study may explain the lower MACE rate in this study compared with PRASFIT-ACS (0% vs. 5%) [9]. Overall, prasugrel responsiveness was not influenced by the distribution of ACS type, which was equally proportioned in the present Switch Study cohort but not in the STENT-Registry or PRASFIT-ACS. Nonetheless, there are distinct differences in study design, size of the population, and duration between the STENT-Registry or PRASFIT-ACS and the current study that limit the direct comparison and/or extrapolation of study data.

The switch from clopidogrel to prasugrel more than halved patients’ HPR rates and can be seen to generate a narrower distribution curve, which may indicate tighter control of platelet activity. The mean PRU was successfully reduced after the switch regardless of gender and ACS characterization. Interestingly, PRU reduction was not statistically significant among patients with UA (*p* = 0.056). The smaller sample size of UA patients relative to the entire group of patients with myocardial infarction may have led to this observation. Only 3.0% of study subjects were found to have HPR (per the protocol definition) at Week 4, leading us to adopt the more stringent post-switch HPR definition (PRU > 208) in the exploratory analysis. Even at the more stringent level, only 10% of the study population had Week 4 PRU > 208, which was associated with HPR at baseline, age ≥ 65 and BMI ≥ 25 (Fig. 3b). A recent Japanese study suggested that HPR (PRU > 208) is independently associated with major adverse cardiac and cerebrovascular events (MACCE) and stent thrombosis [21]; in this study, however, only two MACEs (non-fatal MIs) occurred, and both patients having non-fatal MIs were non-HPR with prasugrel (at Week 4). These findings reflect that HPR is multifactorial and that though patient’s PRU levels are informative of efficacy, physicians should still weigh in patient’s overall conditions (e.g., disease characterization, complexity of coronary artery lesions, bleeding risks, and side effect) to personalize a well-balanced anti-thrombotic treatment.

Throughout study Period 1 and 2, both the occurrences of MACE (1.0%); TIMI major bleeding (2.0%); and BARC 2, 3, and 5 bleeding (7.9%) were relatively low, accompanied by acceptable TIMI minor (6.4%) and non-major, non-minor clinically relevant bleeding (3.0%) among the prespecified subgroups (HPR vs. non-HPR at Week 4; *p* > 0.05). These were comparable to bleeding outcomes in PRASFIT-ACS, including TIMI major bleeding (1.9%); non-major, non-minor clinically relevant bleeding (4.2%); and TIMI minor bleeding (3.9%); whereas rates were lower in the Switch Study in terms of BARC 2, 3, and 5 bleeding (8.4% vs 17.0%) [22]. One patient experienced a TEAE leading to death, but on adjudication was found not to be related to treatment (i.e., car accident). Other AEs throughout study Period 1 and 2 were mostly gastrointestinal or cardiac in origin, among which serious events included gastrointestinal hemorrhage and myocardial infarction.

Despite the lower prevalence of HBR cases in our study cohort compared with prior studies [9], we observed that numerically there was no significant difference in bleeding events (both TIMI major and BARC 2, 3 and 5 bleeding) between HBR and non-HBR patients at the end of 48-week safety evaluation. Of note, the relatively lower HBR rates in this study was driven by the protocol design to exclude patients with high bleeding tendencies (e.g., severe hepatic disease; ESRD; hemoglobin < 10.5 g/dL; prior history of ICH; a recent TIA or ischemic stroke). Nevertheless, the result may still imply that maintenance dose of prasugrel (3.75 mg), when administered to HBR patients, did not invoke high bleeding risk as was expected; such observation deserves to be confirmed with a more robust comparison in future studies.

Given the high prevalence of *CYP2C19 *loss-of-function carriers in East Asians, this study provides meaningful snapshots of pharmacodynamic responses of both prasugrel and clopidogrel in Taiwanese patients with ACS treated with PCI. The study proved that maintenance dose of prasugrel delivers significant PRU reduction after a switch from clopidogrel, and at the same time preserves the modest safety profile as revealed in the 48-week observation. This provides a choice for Taiwanese physicians who face the dilemma to balance between high ischemic (e.g., multivessel or type C lesions) and bleeding (e.g., ARC-HBR) risks, as well as other conditions (e.g., adverse drug reaction, *CYP2C19* gene polymorphism, and compliance). Partly concordant with the post hoc risk factor analysis performed in PRASFIT and PRASFIT-Practice I and II studies [23, 24], we found that patients of female sex, older age, and lower BMI demonstrated higher pharmacological responsiveness to prasugrel. However, to establish the long-term benefit of this regimen among Taiwanese patients, investigating its effects in the real-world setting may be a feasible approach. Of note, the study was not specifically designed to prove strategies of escalation or de-escalation of P2Y12 inhibitors, and the results are not indicative of the outcome or benefit of such strategies [15, 25–28]; more studies are warranted to clarify the benefit of escalation and de-escalation in Taiwanese ACS population.

## Limitations

As a result of the study design, our safety observation only started at the point of switching until the end of treatment (i.e., 12 months). However, pre-switching safety outcomes have been included in our analysis for the purpose of comprehensiveness. Several previous studies reported demographic factors that may be predictive of P2Y12 inhibitor antiplatelet effects [29–31], but these are heterogeneous and difficult to compare with the present study. An additional limitation is that although the proportion of female patients and the prevalence of comorbid diseases in the present study were not notably different from those described in published reports [20, 23], the sample size of the current study prevents detailed subanalysis of patients in these groups. Further, the study did not have a control arm and did not contain data on the ethnic subgroups of the Taiwanese population (e.g., Han, Hakka, and aborigines).

## Conclusions

Switching to prasugrel induced a significant PRU reduction beyond that achieved by maintenance dose of clopidogrel (75 mg) while maintaining a good safety profile and high compliance. Similar to PRASFIT-ACS, the current results suggest that switching to a maintenance dose of prasugrel (3.75 mg) shows good long-term reduction of platelet reactivity across patient populations, without evidently increasing the risk of excessive bleeding and inconsistent antiplatelet effects in post-PCI patients with ACS.

## Supplementary Information

Below is the link to the electronic supplementary material.

Supplementary file1 (DOCX 193 kb)
