# Supplementary material for: Prasugrel switching from clopidogrel after percutaneous coronary intervention for acute coronary syndrome in Taiwanese patients: an analysis of safety and efficacy
Source: Cardiovasc Interv Ther. 2021 Apr 4;37(2):269–78. doi: 10.1007/s12928-021-00771-w (PMC8926957; doi:10.1007/s12928-021-00771-w)
Supplement: Supplementary file 1 — Supplementary file1 (DOCX 193 kb) [file 12928_2021_771_MOESM1_ESM.docx]

**Prasugrel switching from clopidogrel after percutaneous coronary intervention for acute coronary syndrome in Taiwanese patients: An analysis of safety and efficacy**

**Authors**

Ping-Yen Liu^1,2^, Cheng-Huang Su^3,4^, Feng-Yu Kuo^5^, Wen-Lieng Lee^6^, Yi-Chih Wang^7^, Wei-Shiang Lin^8^, Pao-Hsien Chu^9^, Tse-Min Lu^10, 11^, Ping-Han Lo^12^, Cheng-Han Lee^1^, Wei-Ren Lan^5^, Chien-Lung Huang^13^, Shuji Tsukiyama^14^, Wei-Chen Yang^15^, Li-Chung Cheng^15^, Virginia Rafael^16^, Christian Nikolajsen^16^, Wei-Hsian Yin^13,11^

**Affiliations**

^1^ Department of Cardiology, National Cheng Kung University Hospital, College of Medicine, National Cheng Kung University, Tainan, Taiwan

^2^ Institute of Clinical Medicine, College of Medicine, National Cheng Kung University, Tainan, Taiwan

^3^ Cardiovascular Center, Departments of Internal Medicine and Medical Research, Mackay Memorial Hospital, Taipei, Taiwan

^4^ Mackay Medical College, New Taipei City, Taiwan

^5^ Division of Cardiology, Department of Medicine, Kaohsiung Veterans General Hospital, Kaohsiung, Taiwan

^6^ Division of Interventional Cardiology, Cardiovascular Center, Taichung Veterans General Hospital, Taichung, Taiwan

^7^ Division of Cardiology, Department of Internal Medicine, National Taiwan University College of Medicine and Hospital, Taipei, Taiwan

^8^ Division of Cardiology, Tri-Service General Hospital, National Defense Medical Center, Taipei, Taiwan

^9^ Division of Cardiology, Department of Internal Medicine, Chang Gung Memorial Hospital at Linkou, Chang Gung University College of Medicine, Taoyuan, Taiwan

^10^ Division of Cardiology, Department of Medicine, Taipei Veterans General Hospital, Taipei, Taiwan; Healthcare Center, Taipei Veterans General Hospital Taipei, Taiwan

^11^ Faculty of Medicine, National Yang Ming University, Taipei, Taiwan

^12^ Division of Cardiology, Department of Internal Medicine, China Medical University Hospital, China Medical University, Taichung, Taiwan

^13^ Division of Cardiology, Heart Center, Cheng Hsin General Hospital, Taipei, Taiwan

^14^ Daiichi Sankyo Co. Ltd.

^15^ Daiichi Sankyo Taiwan Ltd.

^16^ Linical

**Corresponding author**

Wei-Hsian Yin

Address: Division of Cardiology, Heart Center, Cheng Hsin General Hospital, 45, Cheng Hsin Street, Pai-tou District, Taipei 112, Taiwan

Email address: yinwh88@gmail.com

Fax number: +886 28261242

Telephone number: +886 28264400 (ext. 3465)

**SUPPLEMENTARY DATA**

**Figure S1.** Study design. BID, twice daily; ET, end of treatment; LD, loading dose; MD, maintenance dose; PCI, percutaneous coronary intervention; PRU, P2Y12 reaction unit; Tx, treatment; V, visit.


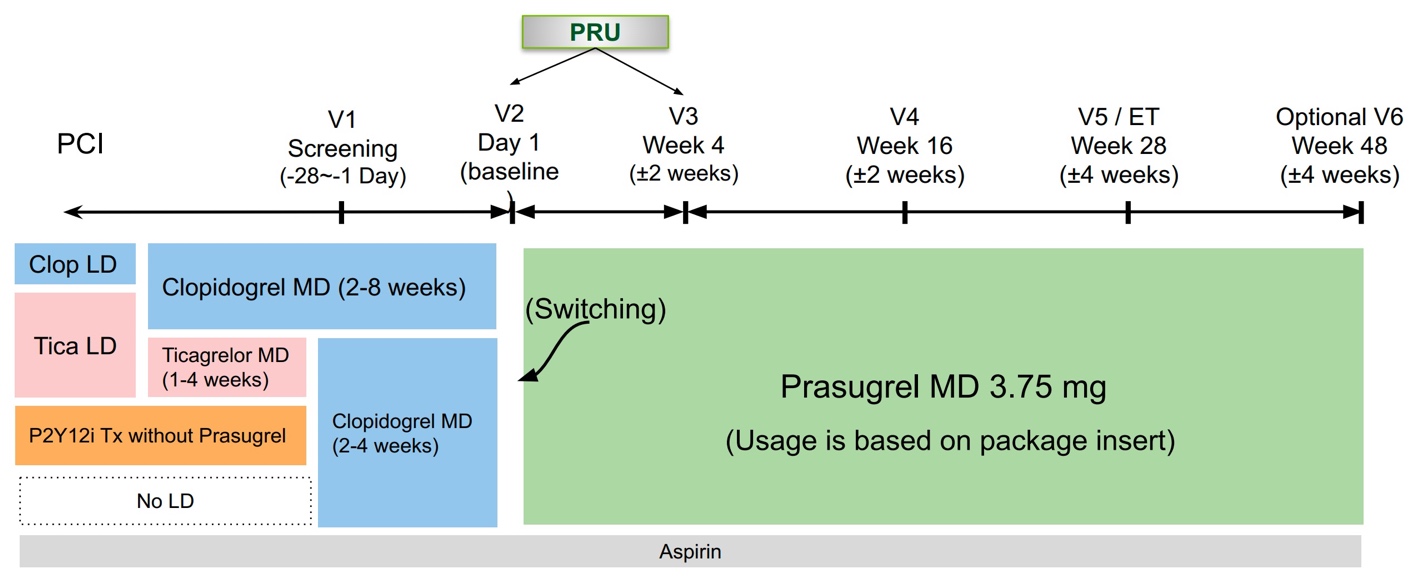


**Table S1.** Bleeding events at a glance: HPR, MACE, and bleedings.

|  | **Baseline**  **(Pre-treatment)** | **Week 4**  **(Treatment-emergent)** | **Week 48**  **(End of treatment)** | **p value*** |
| --- | --- | --- | --- | --- |
| **Efficacy endpoint, n (%)** |  |  |  |  |
| Number of patients | 203 | 200 | – | – |
| HPR (PRU >235) | 23 (11.3) | 6 (3.0) | – | <0.001 |
| HPR (PRU >208) | 48 (23.5) | 20 (10.0) | – | <0.001 |
| **Safety endpoint, n (%)** |  |  |  |  |
| Number of patients | 203 | 203 | 203 | – |
| Any major bleeding | 0 | 0 | 4 (2.0) | – |
| Any minor bleeding | 0 | 0 | 13 (6.4) | – |
| Any clinically relevant bleeding | 3 (1.5) | 1 (0.5) | 6 (3.0) | – |
| Any MACE | 0 | 0 | 2 (1.0) | – |
| BARC 2  BARC 3, 5  BARC 2,3,5 | 0  0  0 | –  –  – | 12 (5.9)  5 (2.5)  16 (7.9) | –  –  – |

*Based on McNemar's test.

Abbreviations: HPR, high on-treatment platelet reactivity; n, number of patients; MACE, major adverse cardiovascular events; PRU, P2Y12 reaction unit.

**Table S2.** Relationship of selected prognostic factors and HPR (PRU >208) after switching to prasugrel. (Safety population, N=203)

| **Prognostic factors** | | **n** | **Event (%)** | **Adjusted OR (95% CI)** | **p value** |
| --- | --- | --- | --- | --- | --- |
| HPR (PRU >208) at baseline | | |  |  | <0.001 |
|  | No | 156 | 4 (2.6) | Reference |  |
|  | Yes | 47 | 16 (34.0) | 18.99 (5.52 to 65.10) |  |
| Age | |  |  |  | 0.048 |
|  | <65 years | 130 | 7 (5.4) | Reference |  |
|  | ≥65 years | 74 | 13 (17.8) | 3.24 (1.01 to 10.45) |  |
|  |  |  |  |  |  |
| BMI | |  |  |  | 0.022 |
|  | <25 | 80 | 4 (5.0) | Reference |  |
|  | ≥25 | 123 | 16 (13.0) | 4.75 (1.25 to 18.04) |  |
| Lipid disorder status | | |  |  | 0.168 |
|  | No | 54 | 9 (16.7) | Reference |  |
|  | Yes | 149 | 11 (7.4) | 0.44 (0.14 to 1.41) |  |

In this multivariate logistic regression model, factors were selected using the stepwise selection procedure (with a probability of 0.2 to determine the entering and staying of variables in the model) from the following list of possible prognostic factors: age, sex, BMI, diabetes status, compliance, and lipid disorder status.

R-squared: 0.2019

Abbreviations: BMI, body mass index; HPR, high on-treatment platelet reactivity; n, number of patients; OR, odds ratio; PRU, P2Y12 reaction unit.
